# Supplementary material for: The Novel Mnk1/2 Degrader and Apoptosis Inducer VNLG-152 Potently Inhibits TNBC Tumor Growth and Metastasis
Source: Cancers (Basel). 2019 Mar 3;11(3):299. doi: 10.3390/cancers11030299 (PMC6468747; doi:10.3390/cancers11030299)
Supplement: Supplementary file 1 [file cancers-11-00299-s001.pdf]

Supplementary Materials: The Novel Mnk1/2 Degrader and Apoptosis Inducer VNLG-152 Potently Inhibits TNBC Tumor Growth and Metastasis

Senthilmurugan Ramalingam, Vidya P. Ramamurthy, Lalji K. Gediya1, Francis N. Murigi, Puranik Purushottamachar, Weiliang Huang, Eun Yong Choi, Yuji Zhang, Tadas S Vasaitis, Maureen A. Kane, Rena G. Lapidus and Vincent C. O. Njar

The following are available online at www.mdpi.com/2072-6694/11/3/299/s1, Synthesis of VNLG-152R and its enantiomers, VNLG-152-biotin, molecular modeling conditions, chromatograms for VNLG-152R and its enantiomers and pharmacokinetics methods.

General Synthetic Chemistry Procedures:

All the materials listed below were of research grade or spectrophotometric grade in the highest purity commercially available from Sigma-Aldrich. Column chromatography was performed on silica (230-400 mesh, 60 Å) from Silicycle. Silica gel plates (Merck F254) were used for thin layer chromatography (TLC) and were developed with mixtures of ethyl acetate (EtOAc)/Petroleum ether or CH_2Cl_2/methanol (MeOH) unless otherwise specified and were visualized with 254 and 365 nm light and I2 or Br2 vapor. Petroleum ether refers to light petroleum, bp 40–60 °C. 1H and 13C NMR spectra were recorded in $CDCl_3$ or DMSO-d_6 using a Brüker 400 MHz NMR instrument and chemical shifts are reported in ppm on the δ scale relative to tetramethylsilane. High resolution mass spectra were obtained on Bruker 12 T APEX-Qe FTICR-MS instrument by positive ion ESI mode by Susan A. Hatcher, Facility Director, College of Sciences Major Instrumentation Cluster, Old Dominion University, Norfolk, VA. Melting points (mp) were determined with Fischer Johns melting point apparatus uncorrected. Purities of the compounds were determined by Waters UPLC BEH C18 1.7µl, 2.1 × 50 mm column using a solvent gradient system of ammonium acetate buffer/acetonitrile/methanol (100→0, 0→100 and 0→10 respectively) over a period of 10 min. The purities of all final compounds were determined to be at least 95% pure by a combination of UPLC, NMR and HRMS.

1. Synthesis of (+) 4-Imidazolyl-(4′-fluoro(phenyl)-(E)-retinamide (VNLG-152R):

The synthesis of VNLG-152R is outlined in Scheme S1 below. VN/14-1 was synthesized as we previously described [1] and was then treated with commercially available 4-fluoroaniline in the presences of 1-hydroxy benzotriazole (HOBT) and dicyclohexylcarbodiimide (DCC) in dimethyl formamide at room temperature.

Scheme S1: Synthesis of VNLG-152R

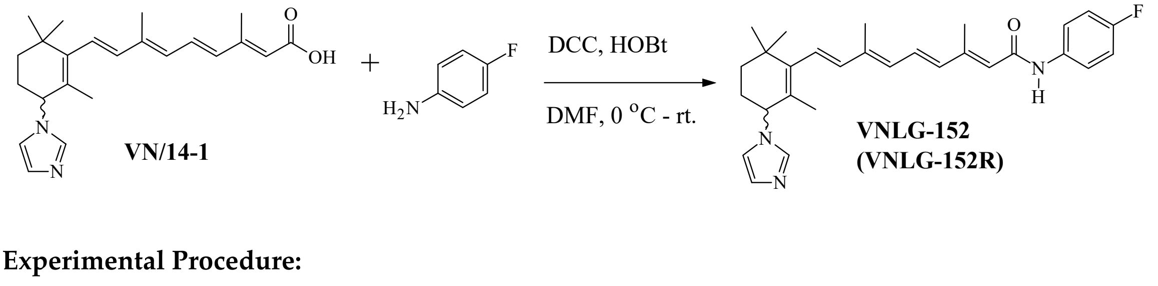

Experimental Procedure:

(+) 4-Imidazolyl-(4′-fluoro(phenyl)-(E)-retinamide (VNLG-152R):

VN/14-1 (1.00 mmol), amine (1.2 mmol), and 1-hydroxy benzotriazole (HOBT) (1.2 mmol) were dissolved in dry DMF (5 mL) and cooled to 0 °C. Dicyclohexylcarbodiimide (DCC), (1.2 mmol) was

added, and the mixture as allowed to warm to room temperature. After stirring for approximately 18 hours, the precipitated dicyclohexyl urea was filtered off and washed with 2 mL of DMF. The filtrate was added drop wise in stirring ice water and the precipitate formed was filtered under vacuum. The vacuum dried precipitate was adsorbed on silica by dissolving in dichloromethane: methanol (8:2) and evaporated under vacuum to obtained dried adsorbed crude product. The crude product which was purified by FCC (silica gel, CH_2Cl_2/EtOH/Et_3N, 9:1:0.2, v/v/v) to give 0.98 mg of desired retinamide, yield = 68%, Mp = 114-115 °C. 1H NMR ($CDCl_3$): δ 1.10 (s, 3H, 16-CH_3), 1.11 (s, 6H,17-CH_3), 1.59 (s, 3H, 18-CH_3), 2.01 (s, 3H, 19-CH_3), 2.41 (s, 3H, 20-CH_3), 4.53 (m, 1H, C4), 5.84 (s, 1H, C14), 6.15-6.31(m, 4H, C7, C8, C10, C12-Hs), 6.91 (m,1H, 4'-H), 6.97 (s, 2H, Ar-H), 7.07 (s, 1H, 4'-H), 7.50 (m, 2H, Ar-H), 7.53 (s, 1H, 5'-H),7.76 (s, 1H, NH). ^{13}C NMR (100 MHz, $CDCl_3$) δ 164.6, 154.1, 146.9, 145.2, 139.5, 137.4, 136.5, 132.3, 131.1, 130.4, 130.2, 128.4, 125.7, 124.5, 121.7, 120.9, 118.5, 115.7, 53.4, 36.5, 31.4, 29.0, 27.7, 24.9, 20.8, 18.8, 12.7. HRMS (ESI) calcd for $C_{29}H_{34}FN_3ONa^+$, 482.2578. Found: 482.2563.

2. Synthesis of VNLG-152 Biotin (VNLG-152R-B)

The synthesis of VNLG-152R-B is outlined in Scheme S2 below. The desired secondary amine (VNFM-89) was prepared from commercially available 4-fluoroaniline and 2-bromoethyacetate via SN2 type reaction [2] Coupling of VNFM-89 with VN/14-1 [1] gave the amide (VNFM-90). Basic hydrolysis of the ester group in VNFM-90 gave amide VNFM-91. Reacting the hydroxyl group of amine with biotin using N-ethyl-N-dimethylaminopropylcarbodiimide hydrochloride (EDC) and a catalytic amount of dimethylaminopyridine (DMAP) gave VNLG-152-B (VNFM-1-92)

Scheme S2: Synthesis of VNLG-152-B

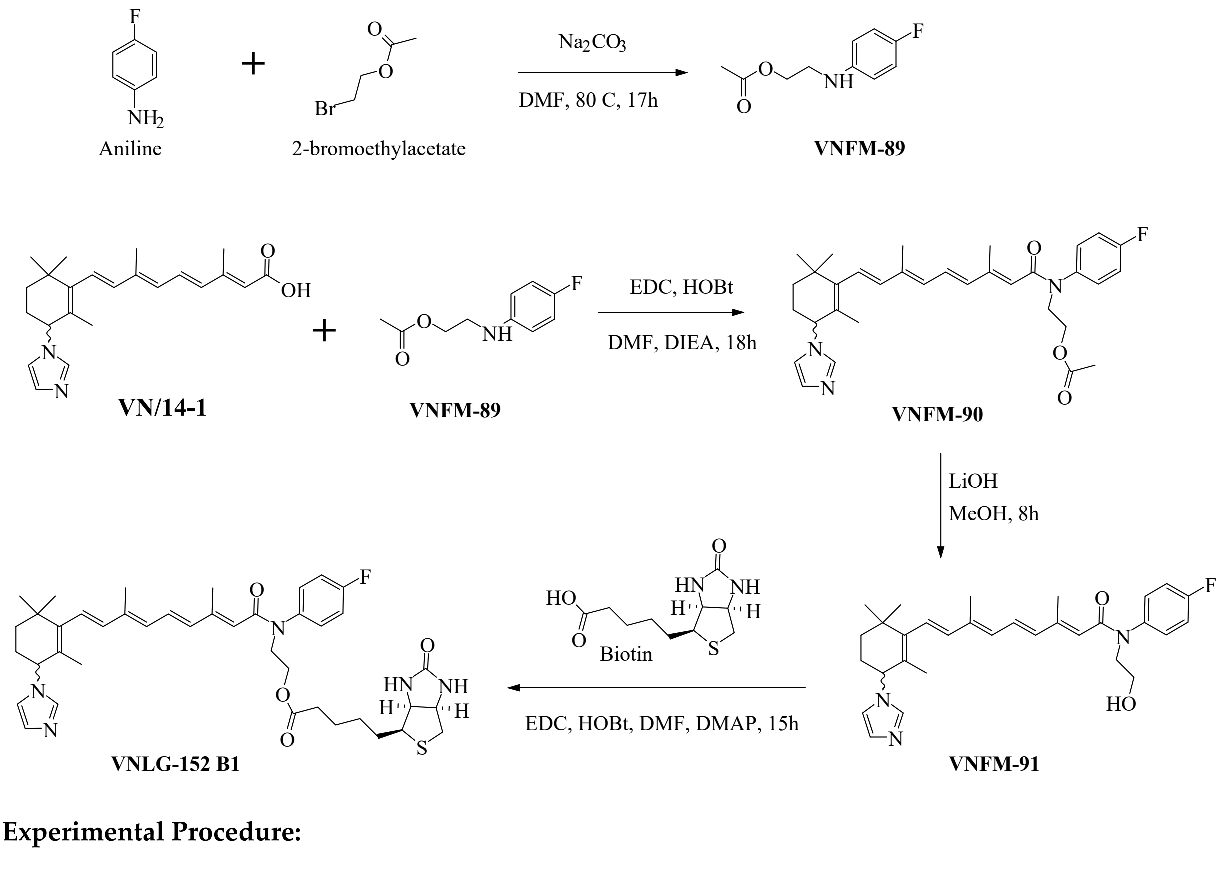

Experimental Procedure:

2-((4-Fluorophenyl)amino)ethyl acetate (VNFM-89)1

Sodium carbonate (1.12 g, 10.6 mmoL) was added to a solution of 4-fluoroanilie (0.59 g, 0.50 mL, 5.28 mmoL) and 2-bromoethylacetate (0.88 g, 0.58 mL, 5,28 mmoL) in DMF (4 mL). The reaction mixture was stirred at 80 °C for 17 h. The reaction mixture was allowed to cool to room temperature,

diluted with water (40 mL) and extracted with EtOAC (2 × 40 mL). The organic extract was washed with water (2 × 30 mL), brine (30 mL), dried over Na_2SO_4, filtered and concentrated in vacuo to yield a crude product. The crude product was purified by flash chromatography (20% EtOAC/petroleum ether) to yield VNFM-89 (0.32 g, 1.32 mmol, 31%) as a white solid. Mp 40–42 °C; Rf 0.33 (30% EtOAC/PE); 1H NMR (400 MHz, $CDCl_3$) δ 6.90 (t, J = 8.7 Hz, 2H), 6.60–6.54 (m, 2H), 4.27 (t, J = 5.4 Hz, 2H), 3.79 (s, 1H), 3.35 (t, J = 5.4 Hz, 2H), 2.09 (d, J = 3.2 Hz, 3H).

2-((2E,4E,6E,8E)-9-(3-(1H-Imidazol-1-yl)-2,6,6-trimethylcyclohex-1-en-1-yl)-N-(4-fluorophenyl)-3,7-dimethylnona-2,4,6,8-tetraenamido)ethyl acetate (VNFM-90) [3]

To a 10 mL round-bottom flask, VN/14-1 (0.40 g, 1.10 mmol), VNFM-89 (0.26 g, 1.32 mmol), EDC (0.42 g, 2.20 mmol), HOBt (0.30 g, 2.20 mmol), and DIEA (0.49 g, 0.66 mL, 3.82 mmol) were dissolved in DMF (2 mL). The reaction mixture was allowed to stir at room temperature for 18 h. The reaction mixture was added dropwise to water (30 mL) in a beaker with stirring. A precipitate was formed that was collected by filtration and washed with cold diethyl ether. Purification by flash chromatography on silica using 3% MeOH/CH_2Cl_2 gave VNFM-90 (0.12 g, 0.22 mmol, 20%) as a pale orange oil, Rf 0.75 (5% MeOH/ CH_2Cl_2); 1H NMR (400 MHz, $CDCl_3$) δ 7.48 (s, 7H), 7.15 (m, 2H), 7.08 (m, 3H), 6.94–6.78 (m, 2H), 6.20 – 5.99 (m, 4H), 5.49 (s, 4H), 4.51 (t, J = 4.7 Hz, 1H), 4.26 (t, J = 5.5 Hz, 2H), 3.98 (t, J = 5.5 Hz, 2H), 2.30 (s, 3H), 2.14–2.00 (m, 2H), 1.97 (s, 3H), 1.95 (s, 3H), 1.88–1.77 (m, 2H), 1.55 (s, 3H), 1.54–1.41 (m, 2H), 1.09 (s, 3H), 1.06 (s, 3H), 0.89–0.81 (m, 2H).

2E,4E,6E,8E)-9-(3-(1H-Imidazol-1-yl)-2,6,6-trimethylcyclohex-1-en-1-yl)-N-(4-fluorophenyl)-N-(2-hydroxyethyl)-3,7-dimethylnona-2,4,6,8-tetraenamide (VNFM-91)

LiOH (80 mg, 3.3 mmol) was added to a solution of VNFM-90 (110 mg, 0.2 mmol) in MeOH at 0 °C. The mixture was stirred for 10 min at 0 °C and allowed to warm to room temperature. After 8 h, the reaction was complete, and volatiles were removed in vacuo to give an oily residue. The residue was cooled in an ice bath, the pH was adjusted to 6 by addition of IM HCL, and the aqueous solution extracted with EtOAC (3 × 15 mL). The organic extract was washed with brine (30 mL), dried over Na_2SO_4, filtered and concentrated in vacuo to yield a crude oily product. The crude oil was washed with a mixture of diethyl ether/petroleum ether to yield 80 mg (0.16 mmol, 80%) of VNFM-91 as a pale orange oil which was used in the next step without further purification. Rf: 0.52 (5% MeOH/ CH_2Cl_2); 1H NMR (400 MHz, $CDCl_3$) δ 7.53 (m, 1H), 7.23–7.15 (m, 2H), 7.14–7.06 (m, 3H), 6.96–6.81 (m, 3H), 6.27–6.01 (m, 5H), 5.51 (s, 1H), 4.52 (m, 1H), 3.97–3.90 (m, 2H), 3.83–3.77 (m, 2H), 2.32 (s, 3H), 2.19–2.00 (m, 4H), 1.98 (s, 3H), 1.86 (m, 2H), 1.60 (s, 2H), 1.56 (s, 3H), 1.53–1.44 (m, 2H), 1.36–1.19 (m, 4H), 1.13 (d, J = 4.4 Hz, 2H), 1.10 (s, 3H), 1.07 (s, 3H), 0.92–0.81 (m, 4H).

2-((2E,4E,6E,8E)-9-(3-(1H-Imidazol-1-yl)-2,6,6-trimethylcyclohex-1-en-1-yl)-N-(4-fluorophenyl)-3,7-dimethylnona-2,4,6,8-tetraenamido)ethyl 5-((3aS,4S,6aR)-2-oxohexahydro-1H-thieno[3,4-d]imidazol-4-yl)pentanoate (VNLG-152R-B) [4]

Biotin (155 mg, 0.64 mmol) and HOBt (104 mg, 0.77 mg) were added to a solution of EDC (147 mg, 0.77 mmol) in DMF (1.0 mL) and the mixture was stirred at room temperature until a homogenous solution was formed. In a separate 5 mL flask, DMAP was added to a solution of VNFM-91(80 mg, 0.16 mmol) in DMF (1.0 mL) and stirred until a clear solution was formed. VNFM-91/DMAP mixture was added to the biotin/HOBT/EDC mixture and stirred for 18 hours at room temperature. The reaction mixture was added dropwise to water (20 mL) in a beaker while stirring. A precipitate was formed and was collected by filtration. The crude product was purified by flash chromatography using 5% MeOH/CH_2Cl_2 to afford 47 mg of VNLG-152-B as an orange solid. Mp 80-82; Rf 0.40 (5% MeOH/ CH_2Cl_2); 1H NMR (400 MHz, $CDCl_3$) δ 7.89 (d, J = 7.6 Hz, 2H), 7.71 (m, 3H), 7.39 (t, J=3.6Hz, 2H), 7.31 (t, J=3.6Hz, 2H), 7.22–7.13 (m,3H), 7.13–7.05 (m, 4H), 6.98–6.77 (m, 3H), 6.33–6.00 (m, 6H), 5.49 (s, 1H), 5.22 (s, 1H), 4.88 (m, 1H), 4.65–4.48 (m, 4H), 4.38–4.19 (m, 5H), 4.09–3.91 (m, 5H), 3.35 (s, 1H), 3.20–3.11 (m, 2H), 2.97–2.86 (m, 2H), 2.78–2.69 (m, 2H), 2.30 (s, 3H), 2.25 (t, J = 7.4 Hz, 3H), 2.17–2.06 (m, 2H), 1.97 (s, 3H), 1.88–1.75 (m, 3H), 1.73–1.36 (m, 18H), 1.25 (s, 6H), 1.09 (s,

3H), 1.06 (s, 3H); HPLC Analysis: retention time 3.067 min [flow rate 0.5 mL/Min, Waters column-C18 3.9 × 150 mm, solvent system: Acetonitrile:water (90:10)]

3. HPLC Chiral Separation of VNLG-152R Enantiomers (VNLG-152E1 and VNLG-152E2)

This study was conducted by Averica Discovery Services Inc., Marlborough, MA, USA. The racemic compound, VNLG-152R (3 g) was resolved to provide the two enantiomers, VNLG-152E1 and VNLG-152E2 using the method below (Table S1):

Table S1. Methods for analytical and preparative chromatography (Analytical SFC Method).

Methods for Analytical and Preparative Chromatography (Analytical SFC Method)	
**Column**	4.6 × 100 mm Chiralpak AD-H from Chiral Technologies (West Chester, PA)
**CO_2 Co-solvent (Solvent B)**	Ethanol with 0.1% Isopropylamine
**Isocratic Method**	25 % Co-solvent at 4 mL/min
**System Pressure**	125 bar
**Column Temperature**	40 °C
**Sample Diluent**	Methanol
**(Preparative SFC Method)**	
**Column**	2.1 × 25.0 cm Chiralpak AD-H from Chiral Technologies (West Chester, PA)
**CO_2 Co-solvent (Solvent B)**	Ethanol with 0.1% Isopropylamine
**Isocratic Method**	30% Co-solvent at 80 g/min
**System Pressure**	125 bar
**Column Temperature**	25 °C
**Sample Diluent**	Methanol

The quantities of the retention times, amounts recovered, and enantiomeric excess of the pure enantiomers are presented in Table S2 below

Table S2. The quantities of the retention times, amounts recovered, and enantiomeric excess of the pure enantiomers.

Fraction	Retention Time	Mass Recovered	Enantiomeric Excess (UV350)
Fraction 1 (AV15193–E1)	2.5 min	885.0 mg	99.7%
Fraction 2 (AV15193–E2)	4.1 min	706.8 mg	98.8%

The collected fractions were dried in a rotary evaporator at 40 °C, rinsed with acetonitrile and transferred into the final vial using methanol and dichloromethane. The chromatograms of the racemate, fraction 1 (VNLG-152E1) and fraction 2 (labeled VNLG-152E2) are shown in Figure S1.

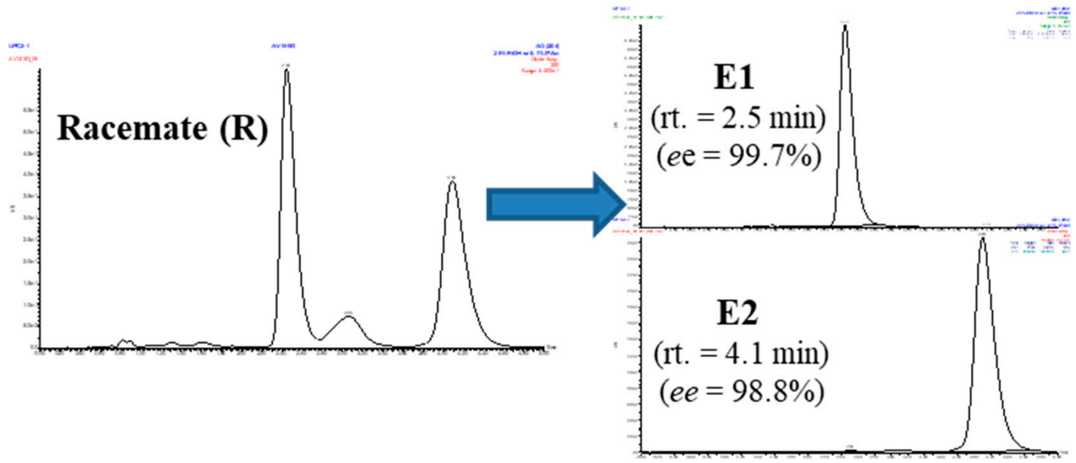

Figure S1. Chromatograms of VNLG-152R and its enantiomers (VNLG-152E1 and VNLG-152E2).

Characterization of the pure enantiomers

As we previously reported for VN/14-1 methylate [1], tentative assignment of the chirality (absolute configuration) at C-4 of the two enantiomers was achieved on the basis of their optical rotations in comparison with the knowledge of the absolute configurations of related (4S)-(+)-4-hydroxyretinal and (4R)-(-)-4-hydroxyretinal [5]

4-R-(-) 4-Imidazolyl-(4'-fluoro(phenyl)-(E)-retinamide(E1): Mp = 114–115 °C. 1H NMR ($CDCl_3$): δ 1.10 (s, 3H, 16-CH_3), 1.11 (s, 6H,17-CH_3), 1.59 (s, 3H, 18-CH_3), 2.01 (s, 3H, 19-CH_3), 2.41 (s, 3H, 20-CH_3), 4.53 (m, 1H, C4), 5.84 (s, 1H, C14), 6.15-6.31(m, 4H,C7,C8,C10,C12-Hs), 6.91 (m,1H, 4'-H), 6.97 (s, 2H, Ar-H), 7.07 (s, 1H, 4′-H), 7.50 (m, 2H, Ar), 7.53 (s, 1H, 5′-H),7.76 (s, 1H, NH). ^{13}C NMR (100 MHz, $CDCl_3$) δ 164.6, 154.1, 146.9, 145.2, 139.5, 137.4, 136.5, 132.3, 131.1, 130.4, 130.2, 128.4, 125.7, 124.5, 121.7, 120.9, 118.5, 115.7, 53.4, 36.5, 31.4, 29.0, 27.7, 24.9, 20.8, 18.8, 12.7.

4-S-(+) 4-Imidazolyl-(4'-fluoro(phenyl)-(E)-retinamide(E2): Mp = 114–115 °C. 1H NMR ($CDCl_3$): δ 1.10 (s, 3H, 16-CH_3), 1.11 (s, 6H,17-CH_3), 1.59 (s, 3H, 18-CH_3), 2.01 (s, 3H, 19-CH_3), 2.41 (s, 3H, 20-CH_3), 4.53 (m, 1H, C4), 5.84 (s, 1, C14), 6.15-6.31(m, 4H,C7,C8,C10,C12-Hs), 6.91 (m,1H, 4'-H), 6.97 (s, 2H, Ar-H), 7.07 (s, 1H, 4′-H), 7.50 (m, 2H, Ar), 7.53 (s, 1H, 5′-H),7.76 (s, 1H, NH). ^{13}C NMR (100 MHz, $CDCl_3$) δ 164.6, 154.1, 146.9, 145.2, 139.5, 137.4, 136.5, 132.3, 131.1, 130.4, 130.2, 128.4, 125.7, 124.5, 121.7, 120.9, 118.5, 115.7, 53.4, 36.5, 31.4, 29.0, 27.7, 24.9, 20.8, 18.8, 12.7.

4. Molecular Modeling Studies

Molecular docking was used to predict the potential binding mode of a lead compound VNLG-152 at MAPK signal-integrating kinase 1 (Mnk1). The ligand was docked into the binding site by sampling its rotational and translational degrees of freedom, and the generated poses were subsequently scored by their estimated free energy of binding. Docking computations were performed on the Mnk1 crystal structure (PDB 2HW6 and 2HW7) [6] The ligand SMILES string was first converted to 2D MOL format by Indigo (EPAM) then to 3D MOL format by OpenBabel [7]. Subsequently, the 3D MOL was converted to PDBQT format by using a ligand preparation script of ADT [8]

The Vina docking algorithm developed by Oleg Trott et al. [9] was used to perform the docking computations. The initial binding center coordinates were derived from the sc-PDB database [10]. Essential hydrogen atoms and Gasteiger charges were added. Non-polar hydrogens, lone-pairs, water molecules and non-standard residues were removed. Position, orientation, and torsions of the ligand molecule were initialized by a random seed. The binding search space was set to 22 Å per dimension, and the exhaustiveness of conformational sampling was set to 8 to balance search thoroughness and simulation time.

5. In vivo Pharmacokinetics (PK) Determination: These studies were Performed by Charles River Laboratories (CRL), Discovery Services, 251 Ballardvale Street, Wilmington, MA 01887, USA.

The PK studies were performed according to the guidelines and approval of CRL Institutional Animal Care and Use Committee (IACUC) under IACUC approval no: P04172013.

A total of sixty-four naïve female CD-1 mice (including 6 spare animals) were received from Charles River Laboratories, Kingston, NY. Following an acclimation period, the animals were assigned to the study based on acceptable health as determined by a staff veterinarian. For dosing, animals were placed into six groups as detailed in the study design table below. All animals were fasted overnight prior to dosing and food was returned after the 4-h post-dose blood collections.

Each animal in Groups 1, 3 and 5 received a single administration of prepared test article by intravenous tail administration at a target dose level of 2 mg/kg and dose volume of 10 mL/kg. Each animal in Groups 2, 4 and 6 received a single administration of prepared test article by oral gavage at a target dose level of 20 mg/kg and at a dose volume of 10 mL/kg. Dosing was performed on August 13, 2015 as detailed in the study protocol and was completed without incident. Dose administration and body weight data are presented in Table S3. Following dosing and at each sample collection time point the animals were observed for any clinically relevant abnormalities.

Table S3. Study Design.

Group	No. of Mices	Test Compound	Dose Level (mg/kg)	Dose Conc. (mg/mL)	Dose Volume (mL/kg)	Dose Vehicle	Route
1	9	(±) VNLG/152 Racemate	2	0.2	10	40% Hydroxypropyl-β-Cyclodextrin (HPβCD)	IV
2	9		20	2	10		PO
3	11	R (−) VNLG/152 E1	2	0.2	10	40% Hydroxypropyl-β-Cyclodextrin (HPβCD)	IV
4	9		20	2	10		PO
5	11	S (+) VNLG/152 E2	2	0.2	10	40% Hydroxypropyl-β-Cyclodextrin (HPβCD)	IV
6	9		20	2	10		PO

Interim blood samples were collected in a leapfrog design from each animal by puncture of a submandibular vein and a terminal blood sample was collected from each animal by intracardiac puncture following euthanasia with CO_2 as outlined in the sample collection table below.

All blood samples were transferred into tubes containing K2EDTA anticoagulant and placed on wet ice immediately after collection until centrifuged at 2200 × g for 10 min at 28 °C to isolate plasma. The resulting plasma was transferred to individual polypropylene tubes in a 96-well plate format and immediately placed on dry ice until storage at nominally 70 °C before transfer to the Test Facility's bioanalysis group for concentration and HPLC analysis. Individual blood collection times are presented in Table S4.

Table S4. Sample Collections.

Dose Groups/ Collection Information	Whole Blood for Plasma	Whole Blood for Plasma
Subgroup A (*n* = 3)	5 min & 1 h post-dose	8 h post-dose
Subgroup B (*n* = 3)	15 min & 4 h post-dose	12 h post-dose
Subgroup C (*n* = 3)	30 min & 6 h post-dose	24 h post-dose
Subgroup D (*n* = 2)	Not applicable	2 h post-dose
Subgroup A (*n* = 3)	15 min & 2 h post-dose	8 h post-dose
Subgroup B (*n* = 3)	30 min & 4 h post-dose	12 h post-dose
Subgroup C (*n* = 3)	1 hr & 6 h post-dose	24 h post-dose
Collection conditions	K_2EDTA	K_2EDTA
Collection route	Submandibular	Intracardiac puncture
Volume/Time point	100 μL	Maximum volume

The plasma samples for Groups 1-6, Subgroups A-C were analyzed for test article concentration using a Research Grade LC MS/MS Assay (RGA-Level 1). The bioanalytical report, including plasma concentration data, were derived accordingly as presented in Figure 3E,F in the manuscript.

Pharmacokinetic parameters were estimated from the plasma concentration-time data using standard non-compartmental methods and utilizing suitable analysis software (Watson 7.2 Bioanalytical LIMS, Thermo Electron Corp). Parameters were estimated using nominal sampling times relative to the start of each dose administration (within an acceptable tolerance limit). Concentrations below the limit of quantification were excluded from the pharmacokinetic analysis.

The area under the plasma test item concentration versus time curve (AUC) was calculated using the linear-log-linear trapezoidal method. When practical, the terminal elimination phase of each concentration versus time curve was identified using at least the final three observed concentration values. The slope of the terminal elimination phase was determined using log linear regression on the unweighted concentration data.

PK Parameters.

Parameter	Description of Parameter
t_{max}	The time after dosing at which the maximum concentration was observed.
C_{max}	The maximum observed concentration measured after dosing.
$AUC_{(0-x)}$	The area under the concentration versus time curve from the start of dose administration to the time after dosing at which the last quantifiable concentration was observed estimated by the linear trapezoidal method.
$AUC_{(0-\infty)}$	The area under the concentration versus time curve from time zero to infinity.
$t_{1/2}$	The apparent terminal elimination half-life.

Equipment and Chromatographic Conditions

HPLC System: Agilent HP 1100
Software: Agilent OpenLab software for analysis and processing
Injection Volume: 20 μL
Flow Rate: 1.8 mL/min
Maximum Absorption, UV (n): 350 nm
Enantiometric Purity: (-) R 52.62% and (*) S 47.38% utilized for racemate calculations
Column: CHIRALPAK® AD-H, 5 microns, 150 mm × 4.6 mm
Mobile Phase A: n-hexane: Isopropanol (90:10) with 0.05% v/v Isopropylamine
Mobile Phase B: Methanol
Isocratic Method:

Time (min)	%A	%B
0	93	7
B	93	7

References

1. Patel, J.B.; Huynh, C.K.; Handratta, V.D.; Gediya, L.K.; Brodie, A.M.; Goloubeva, O.G.; Clement, O.O.; Nanne, I.P.; Soprano, D.R.; Njar, V.C. Novel retinoic acid metabolism blocking agents endowed with multiple biological activities are efficient growth inhibitors of human breast and prostate cancer cells in vitro and a human breast tumor xenograft in nude mice. *J. Med. Chem.* **2004**, *47*, 6716–6729
2. Norman, M.H.; Bo, Y.Y.; Gore, V.K.; Horne, D.; Kaller, M.; Ma, V.V.; Monenschein, H.; Nguyen, T.; Nishimura, N.; Tamayo, N. (2009) Preparation of substituted dihydroisoquinoline derivatives for use as TRP-m8 receptor ligands. WPTO (ed) **2009**, pp 1–180. PCT Int.
3. Mbatia, H.W.; Ramalingam, S.; Ramamurthy, V.P.; Martin, M.S.; Kwegyir-Afful, A.K.; Njar, V.C. Novel C-4 heteroaryl 13-cis-retinamide Mnk/AR degrading agents inhibit cell proliferation and migration and induce apoptosis in human breast and prostate cancer cells and suppress growth of MDA-MB-231 human breast and CWR22Rv1 human prostate tumor xenografts in mice. *J. Med. Chem.* **2015**, *58*, 1900–1914
4. Gortz, V.; Goodby, J.W. Enantioselective segregation in achiral nematic liquid crystals. *Chem. Commun.* **2005**, *26*, 3262–3326
5. Katsuta, Y.; Ito, M.; Yoshihara, K.; Nakanishi, K.; Kikkawa, T.; Fujiwara, T. Synthesis of (+)-(4S)- and (-)-(4R)-(11Z)-4-hydroxyretinals and determination of the absolute stereochemistry of a visual pigment chromophore in te firefly squid, Watasenia scintillans. *J. Org. Chem.* **1994**, *59*, 6917–6921
6. Jauch, R.; Cho, M.K; Jakel, S.; Netter, C.; Schreiter, K.; Aicher, B.; Zweckstetter, M.; Jackle, H.; Wahl, M.C. Mitogen-activated protein kinases interacting kinases are autoinhibited by a reprogrammed activation segment. EMBO J. **2006**, *25*, 4020–4032
7. O'Boyle, N.M.; Banck, M.; James, C.A.; Morley, C.; Vandermeersch, T.; Hutchison, G.R. Open Babel: An open chemical toolbox. *J. Cheminform* **2011** *3*, 33
8. Morris, G.M.; Goodsell, D.S.; Halliday, R.S.; Huey, R.; Hart, W.E.; Belew, R.K.; Olson, A.J. Automated docking using a lamarckian genetic algorithm and and empirical binding free energy function. *J. Comput. Chem.* **1998**, *19*, 1639–1662

9. Trott, O.; Olson, A.J. AutoDock Vina: improving the speed and accuracy of docking with a new scoring function, efficient optimization, and multithreading. *J. Comput. Chem.* **2010**, *31*, 455-461
10. Meslamani, J.; Rognan, D.; Kellenberger, E. sc-PDB: A database for identifying variations and multiplicity of 'druggable' binding sites in proteins. *Bioinformatics* **2011**, 27, 1324–1326

© 2019 by the authors. Licensee MDPI, Basel, Switzerland. This article is an open access article distributed under the terms and conditions of the Creative Commons Attribution (CC BY) license (http://creativecommons.org/licenses/by/4.0/).
